# Association of Polymorphisms of Phase I Metabolizing Genes with Sister Chromatid Exchanges in Occupational Workers Exposed to Toluene Used in Paint Thinners

**DOI:** 10.1155/2015/630296

**Published:** 2015-11-24

**Authors:** Kanu Priya, Anita Yadav, Neeraj Kumar, Sachin Gulati, Neeraj Aggarwal, Ranjan Gupta

**Affiliations:** ^1^Department of Biotechnology, Kurukshetra University, Kurukshetra, Haryana 136119, India; ^2^Department of Microbiology, Kurukshetra University, Kurukshetra, Haryana 136119, India; ^3^Department of Biochemistry, Kurukshetra University, Kurukshetra, Haryana 136119, India

## Abstract

This study investigated genetic damage in paint workers mainly exposed to toluene as it is a major solvent used in paint thinners. Sister chromatid exchange (SCE) assay was used as biomarker of genotoxicity. Blood samples were collected from 30 paint workers and 30 control subjects matched with respect to age and other confounding factors except for exposure to toluene. SCE frequency was found to be significantly higher in paint workers (4.81 ± 0.92) as compared to control individuals (1.73 ± 0.54) (*p* < 0.05). We also investigated influence of polymorphisms of* CYP2E1 *and* CYP1A1m2* genes on SCE frequency. Our results showed that there was significant increase in frequencies of SCE among the mutant genotypes of* CYP2E1* and* CYP1A1m2* as compared to wild genotypes. Our study indicated that long term exposure of toluene can increase genotoxic risk in paint workers.

## 1. Introduction

Organic solvents, a class of chemical genotoxic agents, are mainly used in petrol pumps (benzene, xylene, naphthalene, and 1,2,4-trimethylbenzene), dry cleaning (e.g., tetrachloroethylene), as paint thinners (e.g., toluene, turpentine), and so forth. The exposure of organic solvents can lead to euphoria and hallucinations while high doses may produce life-threatening effects such as convulsions and coma. The long term exposure may lead to cancer risk [1]. Paint workers utilize a variety of chemicals including dyes, solvents (thinners), and finishing chemicals. Toluene is a major solvent used in paint thinners. Given its relatively high volatility, it is predicted that 99 per cent toluene released into the environment is present in the atmosphere. Inhalation of toluene depresses the nervous system. The major effects on humans following acute exposure to high concentrations of toluene include central nervous system dysfunction and unconsciousness. Death can occur in extreme cases. Liver and kidney injury may also occur with exposure to high concentrations. Occupational exposure to moderate levels has been associated with fatigue, sleepiness, headaches, nausea, and decreased manual dexterity and visual perception.

Various kinds of biomarkers have been applied for biological monitoring of exposed population. The examples of such biomarkers include cytogenetic changes (structural and numerical changes in chromosomes, micronuclei formation, sister chromatid exchanges, etc.), somatic mutations, and changes in tumor suppressor genes. Sister chromatid exchanges (SCEs) are interchanges of DNA replication products between sister chromatids at apparently homologous loci, suggested to represent homologous recombination repair of DNA double strand breaks [2, 3]. Biomarkers of susceptibility are indicators of an inherent or acquired ability of an organism to respond to the challenge of exposure to a specific chemical substance. These biomarkers provide an indication of the extent to which an individual may be prone to progress from exposure to developing an adverse health effect.* CYP2E1* and* CYP1A1* genes are mainly involved in metabolism of toluene.* CYP2E1 *gene forms 2 alleles c1 and c2. The genotype c1/c1 was defined as type A, genotype c1/c2 as type B, and homozygote c2/c2 as type C or* CYP2E1* ^*∗*^5B [4]. The* CYP2E1* genetic polymorphism varies significantly among different ethnic groups [5, 6]. The* CYP1A1 *gene is characterized by several polymorphisms, one of which located in exon 7 and consists of an amino acidic exchange (*CYP1A1* ^*∗*^2B or* CYP1A1* m2). The variants of the* CYP1A1 *gene have been extensively studied, especially to evaluate their possible role in DNA damage and cancer promotion [7, 8].

## 2. Materials and Methods

### 2.1. Studied Population

The study was carried out on paint workers (30) and control group consisting of healthy individuals (30) matched with respect to age and other confounding factors except for exposure to toluene. Participants were informed of the objectives of the study. They were asked to sign an informed consent form and each participant was personally interviewed by a standard questionnaire having information related to age, personal medical history, and occupational history of exposure to paint thinners. The research protocol was approved by the Ethical Committee of Kurukshetra University, Kurukshetra, Haryana, India.

### 2.2. Urinary Hippuric Acid Analysis for Internal Toluene Exposure

To assess the toluene exposure in exposed population, the levels of hippuric acid in urine samples of exposed and control subjects were analyzed by colorimetric method [9].

### 2.3. Sample Collection

A total of 5 mL venous blood was collected from each subject in two separate vacutainer tubes containing sodium heparin and dipotassium ethylenediaminetetraacetic acid (EDTA) for lymphocyte culture setup and DNA extraction, respectively. The samples were brought to laboratory in a well-insulated icebox. All exposed individuals have minimum 2 years of working exposure. Blood samples of subjects were collected after finishing of their work shift.

### 2.4. Culture Setup

Short term peripheral blood lymphocytes (PBL) cultures were set up using earlier studied techniques of Moorhead et al. [10] with minor modifications. Cultures were set up in duplicate from whole blood of exposed and control group.

#### 2.4.1. SCE in Peripheral Blood Lymphocytes (PBL)

For sister chromatid exchange (SCE) analysis, whole heparinized blood (0.4 mL) was added to 5 mL of RPMI 1640 culture medium containing 1% L-glutamine, 20% fetal bovine serum, penicillin (100 IU/mL), streptomycin (100 *μ*g/mL), and 2% phytohaemagglutinin. 5-Bromo-2-deoxyuridine in final concentration of 10 *μ*g/mL culture was added after 24 h of incubation. The cultures were incubated for another 48 h at 37°C and ±5% CO_2_. Two drops of colchicines in a final concentration of 0.2 *μ*g/mL was added 45 min prior to the harvesting. The cells were harvested by centrifugation and then treated with hypotonic solution (0.075 M KCl) and fixed in methanol : acetic acid (3 : 1). From a suspension of fixed cells, slides were prepared by the air drying method and stained with Hoechst 33258 and 4% Giemsa stain solution following the method of Perry and Wolff [11]. For calculating the frequency of SCE, 50 well-spread second metaphase stages were analyzed for every participant.

### 2.5. *CYP2E1* ^*∗*^5B Genotyping

The 5-flanking polymorphic site of the* CYP2E1 *gene was analyzed according to protocol of Hayashi et al. [4]. The primers F 5′-CAGTCGAGTCTACAT-TGTC-3′, R 5′ TTCATTCTGTCTTCTAACTG-3′ generated a 410 bp band. PCR products were digested with* Pst1* restriction enzyme and then subjected to electrophoresis on a 2.5% agarose. The genotypes of* CYP2E1 *were classified as homozygous wild (c1/c1) 410 bp, heterozygote (c1/c2) 410, 290, and 120 bps, and homozygous mutant (c2/c2) 290, 120 bps. The digested PCR products were separated by 2.5% agarose gel electrophoresis and were detected by ethidium bromide staining.

### 2.6. *CYP1A1m2* Genotyping

The primers 5′-TTC CAC CCG TTG CAG CAG GAT AGC C-3′ and 5′-CTG TCT CCC TCT GGT TAC AGG AAG-3′ [12] were used for identifying* CYP1A1m2* polymorphism (204 bp). The restriction enzyme* BsrD*I was used to digest PCR product (204 bp) to identify mutant genotypes as later ones generate 149 and 55 bp (for the m2 site) fragments. The restricted products were analyzed by electrophoresis in 3% agarose gel containing ethidium bromide.

### 2.7. Statistical Analysis

Statistical analysis was performed using SPSS software package (version 16.0 for Windows; SPSS, Chicago, IL). All tests were performed in duplicate and results were expressed as means ± SD. Multivariate ANOVA test was used with post hoc analysis for the comparison of frequencies in multiple subgroups among studied population. *p* < 0.05 was considered as the significant level for the statistical analyses.

## 3. Results

The demographic characteristics of studied population have been summarized in Table 1. We collected data from 30 occupationally exposed paint workers and 30 unexposed control subjects. The exposed workers had an average age (years) of 29.77 ± 6.52 while mean age (years) of control subjects was 31.23 ± 6.60. The percentages of smokers, alcohol users, and tobacco users were 20%, 16.7%, and 6.7%. The mean value of exposure duration per day was 6.20 ± 2.93 (hrs).

### 3.1. Urinary Hippuric Acid Assessment

**Figure 1:**
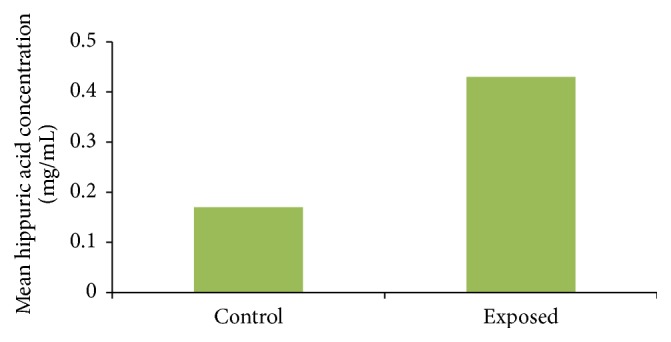
Mean hippuric acid concentration in exposed and control groups.

Mean concentration of hippuric acid in urine samples of exposed subjects (0.43 ± 0.017 mg/mL) was significantly higher than that of control subjects (0.17 ± 0.014 mg/mL) (*p* < 0.05) (Figure 1).

### 3.2. SCE Frequency in Control and Exposed Group

**Figure 2:**
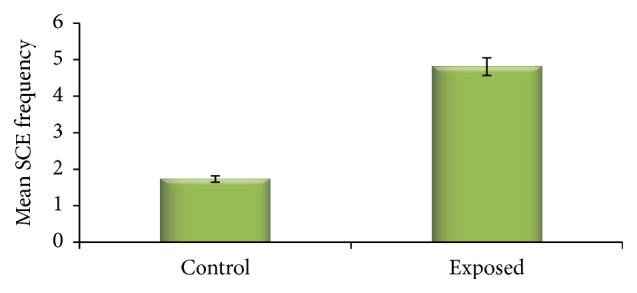
SCE frequency in control and exposed population.

In paint workers mean frequency of SCE was found to be significantly higher (4.81 ± 0.92) than control population (1.73 ± 0.54) (Figure 2).

**Figure 3:**
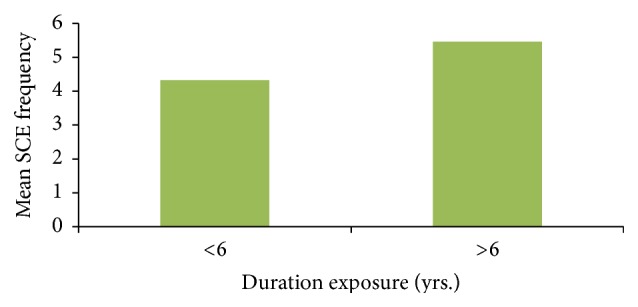
Effect of exposure duration (yrs.) on SCE frequency in exposed population.

Workers having >6 years of exposure duration had significant high SCE frequency (5.46 ± 0.72) compared to individuals having <6 years of exposure (4.32 ± 0.73) (Figure 3).

### 3.3. SCE Frequency by Age and Consumption Habits

Individuals being >40 years were found to have significant higher SCE frequency in exposed and control population (5.83 ± 0.62; 1.95 ± 0.29), respectively (Table 2). There was nonsignificant but high SCE frequency showed in nonsmokers (4.85 ± 0.92); alcoholics (5.05 ± 1.27); and nontobacco users (5.49 ± 0.38) in exposed population.

### 3.4. Influence of* CYP* Genotypes on SCE Frequency

*CYP1A1m2* homozygous mutants showed significant difference in SCE frequency in exposed (5.61 ± 0.66) and control group (2.36 ± 0.00).* CYP2E1* homozygous mutants (*mt/mt*) in exposed subjects (6.11 ± 0.08) and heterozygous mutants (*wt/mt*) in control subjects (1.88 ± 0.45) showed high SCE frequency (Table 3).

Results of linear regression analysis, adjusted for model such as age, gender, exposure, and consumption habits, have been shown in Table 4.

## 4. Discussion

In present study we found that exposed population has significantly high SCE frequency compared to that of control. Popp et al. [13] investigated the frequency of DNA strand breakage and crosslinking and sister chromatid exchange frequency in the lymphocytes of female workers exposed to benzene and toluene. In the female workers significantly raised (*p* < 0.05) SCE values were found. In a study among 25 male public building painters aged between 18 and 62 years in Mexico, increased SCE levels in lymphocytes were found when compared to the same number of sex- and age-matched unexposed controls [14]. Further, SCEs were evaluated in 25 car painters working in different automobile paint shops in Italy and 37 unexposed control subjects (healthy blood donors). The exposed workers had higher frequencies of SCEs than controls (*p* < 0.05) [15]. Contradicting our results, Haglund et al. [16] studied chromosome aberrations and sister chromatid exchanges in Swedish paint industry workers exposed to a mixture of organic solvents, mainly containing xylene or toluene. No difference in the frequency of sister chromatid exchanges (SCEs) was noted in the peripheral lymphocytes of the exposed group of 17 workers and their matched reference group. Our results showed that there was significant increase in frequencies of SCE among the mutant genotypes of* CYP2E1* and* CYP1A1m2* as compared to wild genotypes. Similar to our results, Kezic et al. [17] studied polymorphisms in the genes encoding* CYP1A1, CYP2E1, EPHX1, GSTM1, GSTT1, *and* GSTP1* enzymes in a group of male CSE (chronic solvent encephalopathy) patients (*N* = 97) and controls (*N* = 214). Comparing patients and controls, higher frequencies of the variant ^*∗*^5B allele of the* CYP2E1* gene and of the variant* GSTP1* ^*∗*^ *C* allele were found.

## 5. Conclusion

In our study, we have found that* CYP2E1* and* CYP1A1m2* gene modulate the genotoxic effects in paint workers. The determination of* CYP2E1* and* CYP1A1m2* genotypes status could also be helpful in providing baseline data as an individual marker of susceptibility in exposed population. It is recommended that occupational paint workers are required to train regularly and always be given appropriate personal protective equipment.

## Figures and Tables

**Table 1:** Demographic characteristics of exposed and control population.

Variables	Control	Exposed	*p* value
All (*N*)	30	30	
Age	31.23 ± 6.60	29.77 ± 6.52	0.390
Working exposure per day (hrs)		6.20 ± 2.93	
Duration of exposure (yrs) (*N*) %			
<6		(13) 43.33	<0.05
>6		(17) 56.67	
Smoking			
Nonsmokers	25 (83.3)	24 (80)	0.739
Smokers	5 (16.7)	6 (20)	
Alcohol intake			
Alcohol users	24 (80)	25 (83.3)	0.739
Nonalcohol users	6 (20)	5 (16.7)	
Tobacco intake			
Nontobacco users	26 (86.7)	28 (93.3)	0.389
Tobacco users	4 (13.3)	2 (6.7)	

Student's *t*-test was applied for comparing mean value among control and exposed group. Chi-square test was applied for difference in consumption habits history among studied population. Level of significance was set at *p* < 0.05.

**Table 2:** SCE frequency by age and consumption habits.

Variables	Control	Exposed
	SCE/cell	SCE/cell
	(mean ± SD)	(mean ± SD)
All	1.73 ± 0.54	4.81 ± 0.92^*∗*^
Age (years)		
<30	1.64 ± 0.52	4.47 ± 0.93
30–40	1.83 ± 0.55	5.26 ± 0.70^*∗*^
>40	1.95 ± 0.29^¥^	5.83 ± 0.62^*∗*¥^
Smoking		
Nonsmokers	1.70 ± 0.55	4.85 ± 0.92^¥^
Smokers	1.86 ± 0.47^¥^	4.67 ± 0.99
Alcohol		
Alcohol users	1.70 ± 0.52	4.76 ± 0.86
Nonalcohol users	1.85 ± 0.64^¥^	5.05 ± 1.27^¥^
Tobacco		
Nontobacco users	1.73 ± 0.51^¥^	4.76 ± 0.93
Tobacco users	1.72 ± 0.80	5.49 ± 0.38^¥^

^*∗*^Significant at *p* < 0.05; multivariate ANOVA test was used with post hoc analysis for the comparison in SCE frequency in multiple subgroups among studied population.

^*∗*¥^Significant at *p* < 0.05 + highest mean rank; ^¥^highest mean rank (Kruskal-Wallis *H* test).

**Table 3:** Influence of *CYP *genotypes on SCE frequency.

Genotype	SCE/cell (mean ± SD)			
	Number (*N*)	Control	Number (*N*)	Exposed
*CYP1A1m2*				
* wt/wt*	23	1.66 ± 0.76	16	4.27 ± 0.73
* wt/mt*	6	1.89 ± 0.55	10	5.36 ± 0.74^*∗*^
* mt/mt*	1	2.36 ± 0.00	4	5.61 ± 0.66^*∗*^

*CYP2E1*				
* wt/wt*	25	1.73 ± 0.54	10	4.31 ± 0.64
* wt/mt*	4	1.88 ± 0.45	16	4.80 ± 0.87^*∗*^
* mt/mt*	1	1.01 ± 0.00	4	6.11 ± 0.08^*∗*^

^*∗*^Significant at *p* < 0.05; multivariate ANOVA test was used for the comparison in SCE frequency in multiple subgroups among studied population.

**Table 4:** Influence of *CYP* genotypes on SCE as analyzed by linear regression.

Genotype	*β* ^a^	*R* ^2^	*p* ^b^ value
Control			
*CYP1A1m2*	0.281	0.075	0.144
*CYP2E1*	−0.121	0.012	0.565
Exposed			
*CYP1A1m2*	0.776	0.376	<0.05
*CYP2E1*	0.798	0.335	<0.05

^a^Unstandardised coefficient.

^b^Model *p* value. Regression analysis was used for the differences in SCE frequency adjusted for age, exposure duration, and consumption habits.
